# Fibroblast-derived MT1-MMP promotes tumor progression in vitro and in vivo

**DOI:** 10.1186/1471-2407-6-52

**Published:** 2006-03-06

**Authors:** Wenyue Zhang, Lynn M Matrisian, Kenn Holmbeck, Catherine C Vick, Eben L Rosenthal

**Affiliations:** 1 Department of Surgery, Division of Otolaryngology – Head and Neck Surgery University of Alabama at Birmingham, Birmingham, AL, USA; 2 Department of Cancer Biology, Vanderbilt University School of Medicine, Nashville, TN, USA; 3 National Institute of Dental and Craniofacial Research, Bethesda, MD, USA; 4 Department of Surgery, Division of Gastrointestinal Surgery, University of Alabama at Birmingham, Birmingham, AL, USA

## Abstract

**Background:**

Identification of fibroblast derived factors in tumor progression has the potential to provide novel molecular targets for modulating tumor cell growth and metastasis. Multiple matrix metalloproteases (MMPs) are expressed by both mesenchymal and epithelial cells within head and neck squamous cell carcinomas (HNSCCs), but the relative importance of these enzymes and the cell source is the subject of controversy.

**Methods:**

The invasive potential of HNSCC tumor cells were assessed in vitro atop type I collagen gels in coculture with wild-type (WT), MMP-2 null, MMP-9 null or MT1-MMP null fibroblasts. A floor of mouth mouse model of HNSCC was used to assess in vivo growth after co-injection of FaDu tumor cells with MMP null fibroblasts.

**Results:**

Here we report changes in tumor phenotype when FaDu HNSCCs cells are cocultured with WT, MMP-2 null, MMP-9 null or MT1-MMP null fibroblasts in vitro and in vivo. WT, MMP-2 null and MMP-9 null fibroblasts, but not MT1-MMP null fibroblasts, spontaneously invaded into type I collagen gels. WT fibroblasts stimulated FaDu tumor cell invasion in coculture. This invasive phenotype was unaffected by combination with MMP-9 null fibroblasts, reduced with MMP-2 null fibroblasts (50%) and abrogated in MT1-MMP null fibroblasts. Co-injection of FaDu tumor cells with fibroblasts in an orthotopic oral cavity SCID mouse model demonstrated a reduction of tumor volume using MMP-9 and MMP-2 null fibroblasts (48% and 49%, respectively) compared to WT fibroblasts. Consistent with in vitro studies, MT1-MMP null fibroblasts when co-injected with FaDu cells resulted in a 90% reduction in tumor volume compared to FaDu cells injected with WT fibroblasts.

**Conclusion:**

These data suggest a role for fibroblast-derived MMP-2 and MT1-MMP in HNSCC tumor invasion in vitro and tumor growth in vivo.

## Background

The mass of solid epithelial tumor is composed not only of malignant epithelial cells, but also of fibroblasts, endothelial cells and inflammatory cells that in theory, can contribute to tumor cell growth and metastatic spread. Matrix metalloproteinase (MMP) expression by tumor cells and surrounding stromal cell types is thought to contribute to tumor progression, although the relative importance of fibroblast-derived proteases remains the subject of speculation. Expression of MMPs has been identified in both the epithelial and stromal elements of head and neck squamous cell carcinoma (HNSCC) [1]. Tumor cell interaction with surrounding fibroblasts is thought to generate a microenvironment favorable for tumor growth and invasion and promote MMP expression [2]. Identifying which MMPs are critical for tumor progression remains a prerequisite for development of targeted molecular therapy [3]. Because MMP-2, MMP-9 and MT1-MMP are frequently identified in the stromal tissues within HNSCC [4-6], and expression of these enzymes shown to correlate with patient outcome [1,4,7], we examined the role of fibroblast derived MMP-2, MMP-9 and MT1-MMP. Using an in vitro collagen invasion model and an orthotopic model of tumor growth, we identified a role for MMP-2 and MT1-MMP in tumor growth and invasion.

## Methods

### Cell culture

The FaDu cell line was purchased from American Type Tissue Collection (Manassas, VA). Fibroblasts were isolated from the dermis of late gestation embryos in mice deficient in MT1-MMP[8], MMP-2 [9] and MMP-9 [10]. Mice strains were outbred Black Swiss. Fibroblasts derived from both backgrounds (littermates) were used as control. Fibroblasts were used between passages 2–6 and maintained in DMEM supplemented with 10% (v/v) fetal bovine serum (Mediatech, Herdon, VA) and antibiotics (100 units/ml penicillin and 100 ug/ml streptomycin sulphate, Mediatech). There was one fibroblast preparation for each of the MMP null cell lines that were used in the in vivo and in vitro experiments. There were two wild type fibroblast cell lines isolated and used to confirm the in vitro and in vivo experiments. Cell lines were maintained in DMEM supplemented with 10% (v/v) fetal bovine serum (Mediatech), 2 mM L-glutamine and antibiotics (100 units/ml penicillin and 100 ug/ml streptomycin sulphate). Human recombinant TIMP-1 (Calbiochem, San Diego, CA) and TIMP-2 (R&D Systems) were used for MMP inhibition and serine protease inhibitors leupeptin and epsilon-amino-caproic acid (EACA) were obtained from Sigma (St Louis, MO). E64 (Sigma), deconyl-Arg-Val-Lys-Arg-CMK (Alexis Biochemicals, San Diego, CA), and GM6001 (Calbiochem) were also added to the invasion assay.

### Invasion assays

Type I collagen (0.9 ml) was prepared as described [11] and added to the Costar Transwell dishes (Corning, Inc., Corning, NY). A final concentration of 3.0 mg/ml was obtained. Media was then added to the upper and lower chamber prior to the addition of 2 × 10^5 ^tumor cells/well and/or 5 × 10^5 ^fibroblasts. Consistent with the nature of primary cell culture [12], the fibroblasts grew at different rates relative to wild-type (MMP-2 83%, MMP-9 88%, MT1-MMP 90%). To compensate for the potential negative impact of this growth rate both a 1:2 ratio and a 1:3 ratio of tumor cells to fibroblasts was used for each fibroblast type to compensate for variations in primary culture growth rate [12]. Invasion assays were performed in either FBS or mouse serum-containing medium (derived from either wild-type, MMP-2-/- or MMP9-/- mice). MMP-2 null serum was used for MMP-2 null experiments since MMP-2 is produced in the serum. Failure to do this would allow contamination of the experiment with MMP-2 containing serum. Similarly, MMP-9 null serum was used in MMP-9 null experiments. MT1-MMP is not present in normal serum.

#### Sample fixation and processing

Gels were removed from the Transwell dish after the 6 day incubation period and then placed in 2.7% formaldehyde and embedded in paraffin. Sections (6-um) were cut and stained with hematoxylin and eosin. Tumor cell invasion was assessed by light microscopy in a minimum of four randomly selected sections for each experimental sample. In this assay the number of invading cells was normalized to the total number of cells (surface and invading). The unpaired t-test was used to compare means.

### Immunohistochemical methods

Immunohistochemistry of paraffin embedded sections was performed as previously described [13]. Primary EMMPRIN antibody (Zymed Laboratories, Inc, San Francisco, CA; 1 μg/ml) was used on deparaffinized and rehydrated sections of the type I collagen invasion assay without antigen retrieval. Immunoreactivity was confirmed by assessing the staining with a second EMMPRIN antibody (BD Biosciences, Franklin Lakes, NJ; OX-47 at 2 ug/ml) on consecutive sections.

### In vivo tumor studies

Severe combined immune deficiency (SCID) male mice were obtained (Charles River, Wilmington, MA) and handled in accordance with the institution's IACUC guidelines. Under sterile technique, 4 × 10^5 ^cells FaDu cells with or without fibroblasts (0.015 ml volume) were injected transcervically into the tongue musculature. At this inoculum, FaDu cells will not consistently form tumors in the absence of fibroblasts. To compensate for variations in fibroblast growth rate, the ratio of tumor cells to fibroblasts was assessed at 1:2 and 1:3. Each animal group contained at least 9 animals. Ultrasound measurements were performed using a 55 MHz-based radial probe (VisualSonic, Toronto, Canada) at 10 days under anesthesia and the area of greatest diameter in the coronal plane was found and each tumor size was determined by measuring the greatest coronal cross-section in two dimensions. At 14 days, tumors were resected with the mandible intact, decalcified and processed for histology. Serial sections of the specimen were then cut and stained with hematoxylin and eosin and the tumor volume calculated.

## Results

### In vitro invasion of murine fibroblasts

**Figure 1:**
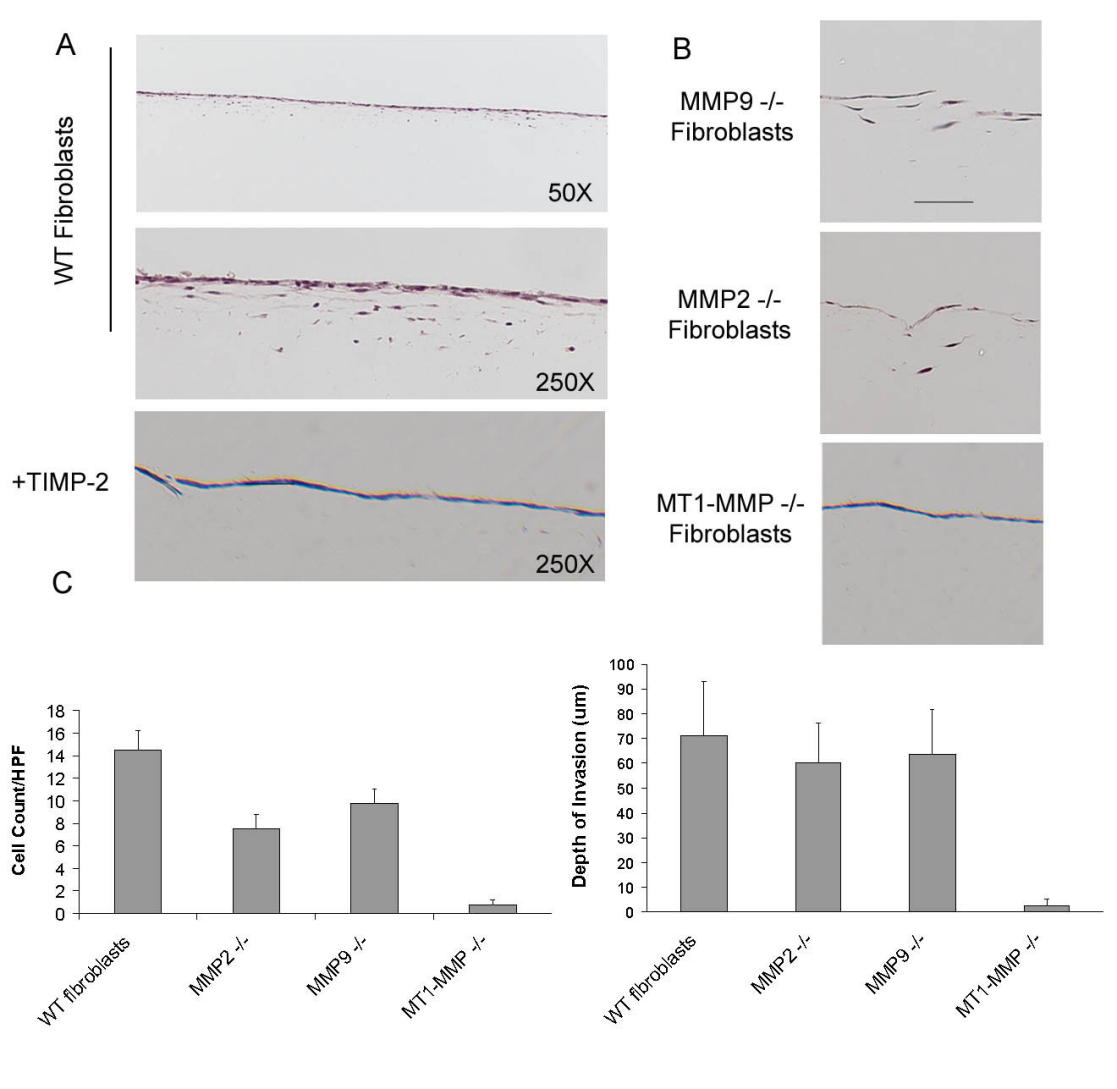
MT1-MMP deficient fibroblasts demonstrate reduced invasion into type I collagen gels. Wild-type (WT) murine fibroblasts derived from the dermis of late-gestation embryos were cultured atop collagen and invasion assessed over a 6 day period in the presence or absence of TIMP-2 (10 nM) added to the culture daily (A). MMP-2 -/- and MMP-9 -/-, but not MT1-MMP -/- cells penetrate type I collagen over the 6 day culture period normalized for total cell number (B); the results are quantified by number of cells invading and depth of penetration (C). Experiments were conducted in DMEM with 10% WT, MMP-2 null or MMP-9 null serum. (Magnification as marked, bar = 100 μm)

Consistent with other reports that have identified the importance of MT1-MMP in remodeling of type I collagen matrices [8,14], we hypothesized that MT1-MMP deficient fibroblasts would be unable to invade type I collagen matrices. Wild-type (WT) fibroblasts were found to invade into type I collagen gels in vitro over the 6 day incubation period (Figure 1A). Invasion was inhibited by tissue inhibitor of metalloproteinase, TIMP-2, and the broad spectrum synthetic MMP inhibitor, GM6001. While MMP-9 and MMP-2 deficient fibroblasts retained an invasive phenotype over the 7 day culture period, MT1-MMP null fibroblasts failed to penetrate the matrix (Fig. 1C). Although fewer cells penetrated the matrix for the MMP-2 and MMP-9 null fibroblasts (p < 0.05), the depth of penetration was the same (Figure 1D). MT1-MMP null fibroblasts had lower penetration into collagen gel compared to WT, MMP-2 null and MMP-9 null fibroblasts.

### Wild-type murine fibroblasts induce MMP-dependent invasion in vitro

**Figure 2:**
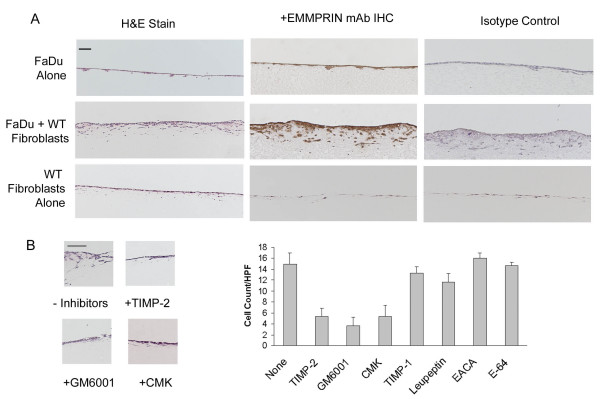
Coculture of FaDu tumor cells and wild-type (WT) fibroblasts induces MMP-dependent tumor cell invasion. WT fibroblasts were cultured in a 2:1 ratio with FaDU HNSCC tumor cells atop Type I collagen for 6 days. EMMPRIN immunoreactivity identified invading tumor cells (A). Protease inhibitors were added to assess the role of MMPs in the invasive program (B). FaDu-WT fibroblast invasion was sensitive to synthetic (GM6001, 20 uM) and endogenous (TIMP2, 10 nM) broad spectrum MMP inhibitors and to furin inhibition (synthetic peptidyl chloromethyl ketones, CMK, 50 uM), but not TIMP-1 (10 nM), serine protease inhibitors (leupeptin, 10 uM; epsilon-amino-caproic acid (EACA), 10 mM) or cysteine protease inhibitors (E64, 10 ug/ml). Bar, 100 μm.

WT murine fibroblasts were cocultured with human tumor cells in a 2:1 or 3:1 ratio with human head and neck squamous cell (HNSCC) tumor cells atop type I collagen over a 6 day incubation period. The fibroblasts induced significant invasion of tumor cells into the matrix (Fig. 2A). A tumor cell:fibroblast ratio of 1:2 or 1:3 was used, but the ratio of cells did not alter the phenotype (data not shown). The high levels of EMMPRIN expression in HNSCC tumors, but not in fibroblasts [13], was used to identify invading tumor cells in the matrix. Synthetic (GM6001, 20 uM) and endogenous (TIMP-2, 10 nM) broad spectrum MMP inhibitors were able to inhibit tumor cell invasion into the matrix (Fig. 2B). Inhibition of furin, which processes MT1-MMP to its active form intracellularly, by CMK (50 uM) blocked invasion, but not addition of TIMP-1 which is a poor inhibitor of membrane anchored MMPs [15]. Serine protease inhibitors (leupeptin, 10 uM; epsilon-amino-caproic acid (EACA), 10 mM) or cysteine protease inhibitors (E64, 10 ug/ml) did not decrease tumor cell invasion.

### MT1-MMP deficient fibroblasts fail to promote in vitro invasion

**Figure 3:**
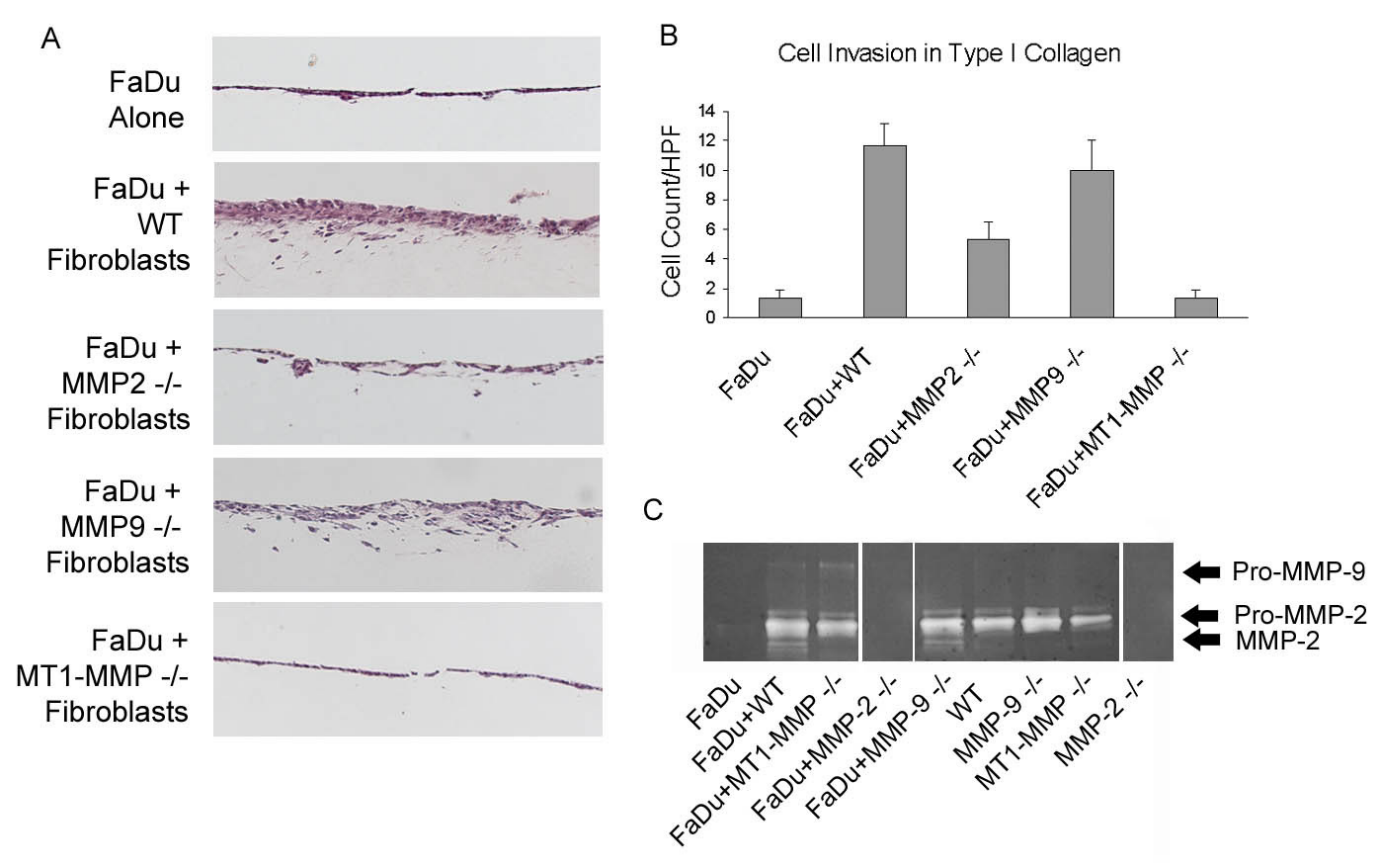
Decreased tumor cell invasion induced by MMP-2 and MT1-MMP null fibroblasts in vitro. The human FaDu HNSCC tumor cell line was cultured alone or in combination with WT, MMP-2 null, MMP-9 null, or MT1-MMP null fibroblasts atop type I collagen for 6 days (A, and quantified in B). Experiments were conducted in DMEM with 10% WT, MMP-2 null or MMP-9 null serum. Gelatin zymography of 18-hour serum free conditioned media from FaDu tumor cells in coculture with fibroblasts atop collagen (2:1 ratio) normalized for cell-number.

To assess whether fibroblast derived MMP-2, MMP-9 or MT1-MMP can promote tumor cell invasion in vitro, the murine fibroblasts were cocultured with human FaDu tumor cells (Fig. 3A and 3B). Because of variations in fibroblast growth rates, cocultures were performed at 2:1 and 3:1 (fibroblast:tumor cell) with similar results (results for 2:1 WT:FaDu and 3:1 MMP null:FaDu cells shown). Unlike the fibroblast invasion assay alone, FaDu tumor cells in coculture with MMP-2 null fibroblasts demonstrated 50% less invasion compared to FaDu cells in coculture with MMP-9 null or WT fibroblasts. This finding suggests a role for MMP-2 in the more complex model of tumor cell invasion. Although FaDu tumor cells express MT1-MMP (data not shown), MT1-MMP null fibroblasts failed to promote FaDu tumor cell invasion. This suggests that localization of this enzyme to fibroblasts induces tumor cell invasion. Incubation of sub-confluent FaDu tumor cells atop type I collagen with serum-free conditioned media from WT, MMP-2 null, MMP-9 null or MT1-MMP null fibroblasts failed to induce FaDU tumor cell invasion (data not shown) over a 7 day incubation period. Analysis of conditioned media obtained from cells cocultured on type I collagen demonstrates that fibroblasts and not tumor cells express proMMP-2 and MMP-9 (Fig. 3C).

### In vivo growth of tumors injected with WT or MMP null fibroblasts

**Figure 4:**
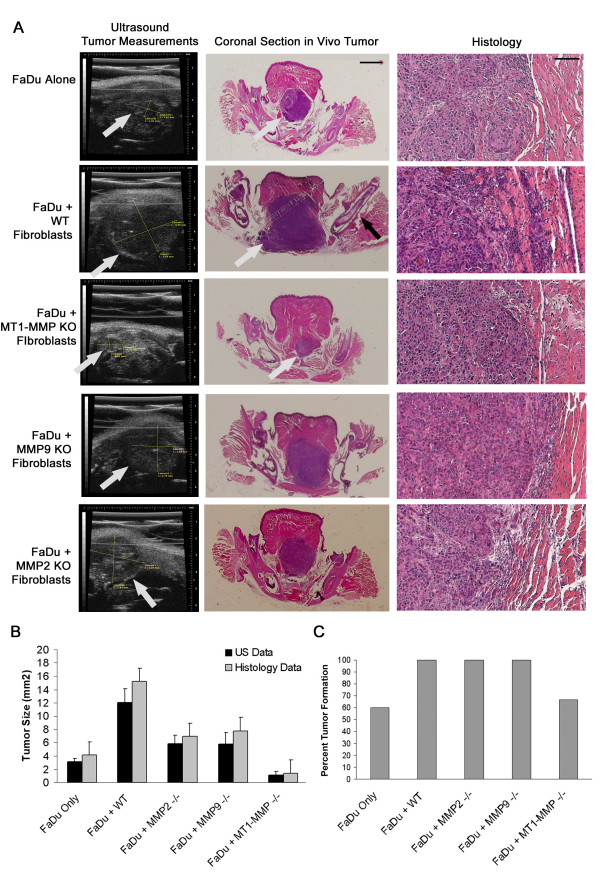
In vivo growth of FaDu cells co-injected with murine fibroblasts. FaDu tumor cells (4 × 10^5 ^cells) were injected transcervically into the oral cavity of SCID mice alone or in combination with WT fibroblasts (8 × 10^5^), MMP-2 null, MMP-9 null or MT1-MMP fibroblasts (8 × 10^5 ^(data not shown) or 1.2 × 10^6 ^cells). Measurements of tumor growth were taken at 10 days by ultrasound examination and at 14 days by histological sectioning of the mandible-tongue complex in the coronal plane (bar is 1 mm in coronal sections or 100 μM in histology) (A). Greatest cross sectional tumor area measurements were compared (B) for both groups. C, percent tumor formation in the orthotopic SCID mouse model over the two week observation period was equal to the number of tumors formed divided by number of mice (one injection per mouse with least 10 mice were injected per group).

To examine the effects of MMP derived fibroblasts in vivo, FaDu tumor cells were injected alone or in combination with WT, MMP-2 null, MMP-9 null, or MT1-MMP null fibroblasts into the oral cavity of SCID mice in ratios of 1:2 and 1:3 (tumor cell:fibroblast). Fibroblasts injected alone did not form tumors. Tumors were assessed at 10 days by ultrasound examination and then at 14 days by histological serial sectioning (Fig. 4A). Assessment of tumor histology, tumor grade, invasive edge, stroma:tumor ratio, or microvessel density did not reveal any consistent differences between groups (data not shown). The FaDu inoculum of tumor cells (4 × 10^5^) in this orthotopic mouse model failed to generate tumors in 40% of mice injected with FaDu cells alone. Consistent with other reports that identify the growth promoting effects of co-injecting fibroblasts with HNSCC cells [16], co-injection of FaDu cells with WT fibroblasts resulted in 100% tumor formation. Interestingly, the MMP-9 null fibroblast had reduced in vivo effect on FaDu tumor growth compared to WT fibroblasts (48% less tumor volume) despite the absence of observed differences for MMP-9 null fibroblasts in previous in vitro experiments. The reduced influence of MMP-2 null fibroblasts on FaDu tumor invasion in vitro and growth in vivo (49% less tumor volume) remained consistent. Smaller tumors were formed when FaDu tumor cells were co-injected with MT1-MMP deficient fibroblasts as compared either MMP-2 null (80% smaller) or MMP-9 null (80% smaller) or WT fibroblasts (92% smaller). However, MMP-2 null and MMP-9 null fibroblasts combined with FaDu tumor cells formed significantly larger tumors than FaDu cells injected alone (p = 0.04 and 0.009, respectively). MT1-MMP remained critical for the positive growth effect of co-injected fibroblasts with reduced tumor size (p = 0.0001, compared with WT and FaDu cells) and incidence of formation. Although MMP-2 null and MMP-9 null fibroblasts formed smaller tumors in vivo, the take rate for these fibroblasts in combination with tumor cells was 100% of injected animals (Fig. 4C). MT1-MMP fibroblasts combined with tumor cells failed to form tumors in 30% of injections, which approached statistical significance when compared to coinjection with WT fibroblasts (p = 0.08).

## Discussion

Recent attempts to understand tumor growth have focused on the surrounding stroma which is considered important in epithelial transformation, cellular invasion and tumor growth [17,18]. The importance of MMPs in tumor cell migration through the extracellular matrix has been established [2,12,19-21], even if the cellular source of the proteases remains uncertain. Our results suggest the importance of fibroblast-derived MT1-MMP and MMP-2 in head and neck squamous cell carcinoma cell invasion in vitro and tumor cell growth in vivo.

The growth promoting effects of fibroblasts in multiple tumor types has been demonstrated [22,23], including in HNSCC cell lines [16]. Indeed, fibroblasts promote growth in MCF7 cells in vivo that can be inhibited with TIMP-2 or synthetic MMP inhibitor [24]. Cocultures of FaDu cells and MT1-MMP null fibroblasts dramatically abrogated the effect of WT fibroblasts in promoting FaDu tumor cell invasion in vitro and tumor growth in vivo suggesting a prominent role for this enzyme. This data is consistent with the importance of membrane type-1 MMP (MT1-MMP) in physiological processes such post-natal murine development [8] and in single cell in vitro invasion [14]. MT1-MMP is expressed in both tumor cells and fibroblasts [1,25].

The significance of MT1-MMP expression in tumor cells has convincingly demonstrated that overexpression of MT1-MMP in tumor cells promotes growth [26]. The data presented here examines the influence of fibroblast derived MT1-MMP during tumor invasion into type I collagen and tumor growth in vivo. Although it is known that MT1-MMP is expressed by the tumor cell (as well the fibroblast), the cell source of this membrane bound protein may determine function. We demonstrated that in an in vitro setting that fibroblast-derived, but not tumor cell derived, MT1-MMP was responsible for type I collagen degradation [27]. Although all in vitro invasion assays were conducted in the presence of serum, additional growth factors were not required to induce invasion when tumor cells were combined with fibroblasts. Indeed, it may be that MMP-dependent cleavage of growth factors combined with reciprocal signaling between tumor cell and fibroblast promotes invasion. The mechanism of MT1-MMP activity in this process requires additional studies to determine whether MT1-MMP expression in fibroblasts disrupts cell-cell signaling, cell growth, tumor cell invasion, and/or promotes matrix degradation.

Our data suggest a role for MMP-2 during in vitro invasion and MMP-9 and MMP-2 during in vivo tumor cell growth. Although it would be more convenient, it is not surprising that more than a single protease enables tumor cell migration through extracellular matrix barriers or growth, particularly in complex models of tumor cell invasion or growth. Indeed, with increasing complexity of the model system, the importance of MMP-2 and MMP-9 became apparent. MMP-2 null fibroblast invasion was comparable to wild-type in the absence of tumor cells, but MMP-2 deficient fibroblasts cocultured with FaDu tumor cells decreased in vitro invasion by almost 50%. Others have failed to identify a role for MMP-2 in a type I collagen in vitro assay assessing endothelial cell invasion [28]. Unlike this study and others that assay invasion using a single cell type [14], we report that in tumor-mesenchymal cell co-culture multiple MMPs may play a role in the invasive program. In the in vivo setting, both MMP-2 and MMP-9 null fibroblasts decreased FaDu tumor volume compared to wild-type fibroblasts, but MMP-2 and MMP-9 null fibroblasts promoted 100% tumor formation. The effect of these two enzymes is likely under represented by this model. While the oral cavity SCID mouse model of HNSCC requires less than 7 days to obtain measurable tumors, providing limited time for in growth of surrounding tissue, it is possible that the elaboration of secreted MMPs by surrounding tissues and the presence of MMP-2 and MMP-9 in the serum prevent formation of a tumor microenvironment devoid of MMP-2 or MMP-9.

Thus, loss of MT1-MMP consistently decreased the invasion or growth promoting influence of fibroblasts in vivo and in vitro and loss of MMP-2 and MMP-9 altered tumor growth with increasing complexity of the model system. Because MT1-MMP is known to activate MMP-2, reduced tumor cell invasion and tumor growth demonstrated in coculture experiments with MT1-MMP null fibroblasts may in part result from a failure to activate pro-MMP-2. However, since loss of MMP-2 produced more subtle phenotypic changes, fibroblast-derived MT1-MMP must have alternate enzymatic targets. Because recent evidence suggests that MT1-MMP (but not MMP-2 or MMP-9) express proteolytic activity that mediates fibroblast migration through type I collagen [14] and MMP-2 and MMP-9 deficient mice develop normally, it is possible that MMP-2 or MMP-9 play a role in more complex models of tumor invasion or growth because they disrupt tumor-stromal signaling rather than matrix degradation. Indeed, there is significant work to suggest a role for both these enzymes in tumor-stromal signaling [2,12].

It is unlikely that that this signaling mechanism involved an alteration in neovascularization. Histological exam of xenografted tumors did not demonstrate differences in microvessel density or histology (Figure 4 and data not shown). These results should be interpreted with caution as the significance of microvessel density remains unclear in HNSCC. In head and neck cancer, microvessel density is not associated with clinicopathological features [29].

It remains unclear why host derived MMPs from the serum or surrounding fibroblasts did not compensate for injected MMP null fibroblasts. This is most likely because of the rapid growth of the tumor over a one week period, not allowing a significant influence from surrounding cells. It is possible that the host MMPs did partially compensate (e.g., the reduced growth rate of MMP-2 and MMP-9 null fibroblasts compared to wild-type fibroblasts cocultured with FaDu cells in vivo represents a host compensation for an otherwise more significant effect). However, despite the potential influence of host MMPs, the tumor volume was still reduced in both experimental groups, suggesting that fibroblast derived MMP-2 and MMP-9 promote tumor cell growth in vivo. Given the results of our in vitro studies, these enzymes may play a complex role in the tumor-stromal signaling, rather than matrix degradation. In other reports, human xenografts in MMP-2 deficient mice demonstrated a 39% reduction in growth, respectively [9]. This is consistent with our decreased growth rate.

The results of these studies suggest that fibroblast derived MT1-MMP promotes tumor cell invasion and growth. There were no morphological differences between in vivo tumors in microvascular density, invasive pattern or stromal density to suggest a possible explanation for the differential rate of tumor growth. Because in vitro results (decreased invasion) may not translate to the phenotype measured in vivo (decreased growth), addressing the mechanism in vitro will not necessarily predict results in vivo. The mechanism by which fibroblast derived MT1-MMP promotes tumor growth is under investigation in our lab. These results have not been previously demonstrated in two cell system or in vivo. The failure of MMP inhibitors in prior phase III clinical trials has been widely considered a failure to understand the complex biology of MMPs in tumor progression [3]. These results suggest that targeted MT1-MMP therapy may limit tumor cell growth and metastasis.

## Conclusion

Collectively, these data suggest a role for fibroblast-derived MMP-2 and MT1-MMP in HNSCC tumor invasion in vitro and tumor growth in vivo.

## Abbreviations

MMP, matrix metalloproteinase; MT1-MMP, membrane-type I MMP, HNSCC, head and neck squamous cell carcinoma; TIMP, tissue inhibitor of metalloproteinase; WT, wild-type; SCID, severe immunodeficient; EMMPRIN, extracellular matrix metalloproteinase inducer; EACA, epsilon-amino-caproic acid.

## Competing interests

The author(s) declare that they have no competing interests.

## Authors' contributions

WZ performed tissue culture and animal studies, CV provide statistical analysis, KH and LM provided study design and data analysis, and ER provided study design and drafted the manuscript. All authors read and approved the final manuscript.

## Pre-publication history

The pre-publication history for this paper can be accessed here:
